# Editorial: Virtual Reality for Sensorimotor Rehabilitation of Neurological Health Conditions Across the Lifespan

**DOI:** 10.3389/fneur.2021.766349

**Published:** 2021-11-25

**Authors:** Carlos Bandeira de Mello Monteiro, Helen Dawes, Judith E. Deutsch

**Affiliations:** ^1^School of Arts, Science and Humanities, University of São Paulo, São Paulo, Brazil; ^2^Department of Sport, Health, Sciences and Social Work, Oxford Brookes University, Oxford, United Kingdom; ^3^College of Medicine and Health, University of Exeter, Exeter, United Kingdom; ^4^Oxford Health BRC, University of Oxford, Oxford, United Kingdom; ^5^Rivers Lab, Department of Rehabilitation and Movement Sciences, School of Health Professions, Rutgers University, Newark, NJ, United States

**Keywords:** sensorimotor rehabilitation, virtual reality, rehabilitation technology, exergames, development of technologies

The National Institutes of Health define, rehabilitation technology as tools that help people recover their function after injury or illness. In recent years, advances in rehabilitation technology have created exciting opportunities and generated significant improvements in the autonomy and quality of life of people with neurological health conditions. Virtual reality (VR) in particular, is a rehabilitation technology that has rapidly risen to prominence and is achieving promising results in improving the sensorimotor function for people with neurological disabilities (1–3). Virtual reality uses interactive simulations created with computer hardware and software to present users with opportunities to perform activities in virtual environments with life-like objects and events. Development of technologies for both the assessment and treatment of persons with neurological health conditions has the potential to either adapt to or target underlying sensorimotor dysfunction and improve body structure, activities, and participation (4).

Given the growing interest in the use of technology in neurological rehabilitation, studies are needed to justify the safe effective use of Virtual Reality in clinical practice. This special issue aimed to collect insightful and multi-disciplinary evidence of the development, testing and application of virtual reality innovations for sensorimotor rehabilitation of neurological health conditions across the lifespan.

Ten papers were published in this Research Topic with contributions to support practice. Espy et al. presents a conceptual framework to guide clinical-decision making for the selection, adaptation, modulation, and progression of virtual reality or gaming when used as a therapeutic exercise modality. The study of Oliveira et al. found benefits in using virtual reality-based exercise in spatial navigation of institutionalized older persons. Cheng et al. investigated performance variability over time during learning of standing postural control tasks in a non-immersive virtual environment in children with cerebral palsy. Benady et al. studied the contribution of vision to locomotion in a dynamic immersive environments to support rehabilitation strategies for neurological disorders associated with gait impairments.

Interestingly, six studies presented the development and use of custom games instead of using non-custom commercial games (Tong et al.; Finley et al.; Al-Sharman et al.; Lubetzky et al.; Da silva et al. and Fluet et al.). Although evidence suggesting that non-custom commercial games can be successfully used in clinical settings, they have limitations such as calibration of a game's difficulty for persons with different abilities, game scores or progress measurements being to generic, lacking specificity in tracking the progress of persons with different abilities, and many require movements that cannot be performed by people with disabilities (5).

Custom games were developed by, Tong et al. who created a ball-pushing task to be used in the HTC VIVE's. Individuals with Phantom limb pain “inhabit” a virtual body (avatar) and the movements of their intact limbs are mirrored in the avatar, providing participants with the illusion that their limbs respond as if they were both intact and functional. They found that repetitive exposure to VR intervention led to reduced pain and improvements in anxiety, depression, and a sense of embodiment of the virtual body.

Finley et al. presented a custom VR game where individuals with Parkinson's disease have to complete a puzzle that consisted of a word with missing letters in the virtual environment. The player had to determine which letters were necessary to complete the puzzle, collect the necessary virtual letters as they floated in 3D space, and then place the letters in the appropriate location. Al-Sharman et al. created a non-immersive VR task to be used with Microsoft Kinect sensor and found improvement in participants with Parkinson's disease when asked to steer a helicopter up and down to collect coins and to avoid specific number of obstacles by moving from sitting to standing and vice versa. Lubetzky et al. created two VR tasks (one using stars on the sky and the other a busy street) with Head Mounted Displays (Oculus Rift) and found significant differences in performance between environments evaluating Postural and Head Control in individuals with unilateral vestibular hypofunction and monaural hearing. Da silva et al. in a study protocol presented two non-immersive custom virtual reality games developed for individuals with disabilities (movehero and moveletrando), both can be used with computer webcam. Fluet et al., presented different studies using a home-based virtual rehabilitation system and a robot assisted virtual rehabilitation to improve paretic hand and arm of persons with chronic stroke. They suggested that persons with stroke may adapt to virtual rehabilitation of hand function differently based on their level of impairment and stage of recovery.

We believe that the studies published in this special issue present research on the benefits of using virtual reality in the rehabilitation for persons with neurological health conditions. It is noteworthy that many authors are integrating game mechanics into their virtual rehabilitation. The research will need to be ongoing to facilitate application to clinical practice.

## Author Contributions

All authors read and approved the final editorial.

## Funding

HD was funded by the Elizabeth Casson Trust and the NIHR Oxford Health Biomedical Research Centre. CM was funded by Fundação de Amparo a Pesquisa do Estado de São Paulo (FAPESP)- Finance Code: 2017/24991-7 and Conselho Nacional de Desenvolvimento Científico e Tecnológico (CNPq). JD was funded by NIH (1R15AG063348-01).

## Conflict of Interest

The authors declare that the research was conducted in the absence of any commercial or financial relationships that could be construed as a potential conflict of interest.

## Publisher's Note

All claims expressed in this article are solely those of the authors and do not necessarily represent those of their affiliated organizations, or those of the publisher, the editors and the reviewers. Any product that may be evaluated in this article, or claim that may be made by its manufacturer, is not guaranteed or endorsed by the publisher.
